# Virulence and transmission characteristic of H3N8 avian influenza virus circulating in chickens in China

**DOI:** 10.1080/21505594.2026.2613516

**Published:** 2026-01-07

**Authors:** Mei Mei, Xuehua Zhang, Qi Wu, Mengcheng Xu, Yongqian Zhao

**Affiliations:** a Institute of Veterinary Immunology and Engineering, Jiangsu Academy of Agricultural Sciences, Nanjing, China; b GuoTai (Taizhou) Center of Technology Innovation for Veterinary Biologicals, Taizhou, China; c Jiangsu Key Laboratory of Food and Safety-State Key Laboratory Cultivation Base, Ministry of Science and Technology, Nanjing, China

**Keywords:** H3N8, avian influenza virus, evolution, virulence, transmission characteristic

## Abstract

Influenza H3N8 viruses have been frequently isolated from chicken farms. However, comprehensive characterization of their virological properties, molecular evolution, virulence, and risk of spillover into mammals remains limited. In particular, little attention has been given to the transmission efficiency of H3N8 avian influenza viruses among chickens and their spillover risk. Here, we systematically characterized H3N8 isolates obtained from asymptomatic chickens through multidisciplinary approaches, including genomic surveillance, receptor binding profiling, and in vivo pathogenicity and transmission assays. All strains showed >98% nucleotide homology with human-infecting strains. Phylogenetic analysis revealed that their internal genes were derived from H9N2, while HA and PB2 genes shared high homology (bootstrap support >98%) with the novel H3N3 virus. All isolates maintained avian-type receptor-binding motifs (HA-Q226/G228) while exhibiting dual α2,3/α2,6-sialic acid binding and robust replication in mammalian cells (peak MDCK titer: 10^7^·^5^ TCID_5__0_/mL). ZJ07 demonstrated exceptional thermostability (HA activity persisting >3 hr at 56°C), while JS13 showed 1.8-fold elevated neuraminidase activity versus controls (*p* < 0.05). *In vivo*, all strains caused subclinical infections with broad tissue tropism in chickens and mice without adaptation, transmitting efficiently among direct-contact poultry. Strikingly, AH12 achieved 100% airborne transmission in chickens. These findings confirm H3N8’s capacity for silent poultry circulation and identify key features conducive to cross-species infection, including dual receptor binding, infection in a mammalian model, and high genetic homology with human strains. The airborne transmissibility of AH12 underscores a heightened spillover risk, necessitating enhanced surveillance and vaccines targeting avian-human interface strains.

## Introduction

Avian influenza virus (AIVs) is influenza A virus which belongs to the orthomyxoviridae family, and consist of eight single-stranded negative sense RNA fragments. AIV is divided into subtypes according to the antigenicity of surface glycoproteins, including 16 hemagglutinin (HA) subtypes (H1-16) and 9 neuraminidase (NA) subtypes (N1-9) [1].

AIVs can infect a wide range of hosts, including chickens, ducks, wild birds, cows, pigs, dogs, cats, horses, humans ect.al. In recent years, more and more avian influenza viruses have been found to be infectious to mammals and pose a threat to the health of humans and other animals. For example, H5N1, H5N6, H7N9, H9N2, H7N4, H10N3, H10N8, H3N8, and H10N5 subtypes have been identified to cause human infections [2–10]. Of which, however, the recent concerns were the two cases of human infection with avian H3N8 virus first reported in China in 2022 [11,12], then a case of human death after infection was happened in Guangdong Province in 2023 [13,14], this has aroused widespread concern around the world due to the viral ability to cross species barriers and cause severe disease in humans [15–17].

H3N8 subtype avian influenza virus is predominantly maintained in wild bird and domestic duck populations, documented cases of cross-species transmission have been reported across multiple mammalian hosts (e.g. equids, canids, swine, pinnipeds, and donkeys) [18–20]. However, the virus was frequently detected in chicken farms and live poultry markets (LPMs) in China in recent years, and resulting in human infection. The human-infection events suggested that H3N8 virus could easily cross species barriers, it not only poses a potential threat to animal and human health but also has the potential to trigger the epidemic of virus in the population. Therefore, the underlying causes and the possibility of reemerging need to be of concern, and long-term and rigorous epidemiological surveillance is essential.

Some animal studies indicated that the H3N8 virus was well adapted in chickens [21–25], but the understanding of the molecular evolution, virulence, and transmission characteristic of the H3N8 virus from poultry is not yet comprehensive. To investigate the virological characteristics of H3N8 viruses, in this study, we focused on the identification and analysis of the H3N8 isolates derived from farmed asymptomatic chickens in eastern China in terms of genetic characteristics, biological activity, pathogenic and transmission characteristic through *in vivo* and *in vitro* experiments. These findings provide important information on the evolution, virulence, and transmission characteristic of the chicken-originated H3N8 virus and provide insights for the surveillance and control of these viruses at the poultry and human interface.

## Materials and methods

### Ethics statement and facility

This study was conducted in strict accordance with the Guidelines for the Care and Use of Laboratory Animals of Jiangsu Province and international regulations on animal welfare. All animal procedures were reviewed and specifically approved by the Committee on the Ethics of Animal Experiments of Jiangsu Academy of Agricultural Sciences (Approval No. IACUC-RE-2022–12-006 for chickens and IACUC-RE-2022–12-007 for mice, Animal License No. SYXK (Su) 2020–0024). No additional approvals from an independent review board or other centers were required for this single-site study. Animals were housed in negative-pressure isolators equipped with HEPA filters under Biosafety Level 2 (BSL-2) conditions, following the institutional biosafety manual.

### Experimental animals

Six-week-old healthy female BALB/c mice were purchased from Yangzhou University (Yangzhou, China) and housed by group in standardized cages with unrestricted access to food and water. Specific-pathogen-free (SPF) embryonated chicken eggs and three-week-old SPF chickens were purchased from Beijing Boehringer Ingelheim Vital Biotechnology Co. LTD.

### Virus isolation and identification

Oropharyngeal swabs were obtained from chickens in poultry farms across Jiangsu, Zhejiang, and Anhui provinces of China in 2022. Viral isolation was performed by inoculating 10-day-old SPF embryonated chicken eggs with 0.2 mL of filtered swab supernatant. Embryos that died within 24 hr post-inoculation(hpi) were discarded. Allantoic fluid was harvested from surviving embryos at 72 hpi and subjected to hemagglutination assay (HA) using 1% (v/v) chicken erythrocytes.

HA-positive samples were processed for RNA extraction (Vazyme, Nanjing, China), followed by reverse transcription into cDNA using the HiScript III RT SuperMix (Vazyme, Nanjing, China) with universal primer 5′-AGCAAAAGCAGG-3′ (Hoffmann et al., 2001). The eight genomic segments (PB2, PB1, PA, HA, NP, NA, M, NS) were amplified via polymerase chain reaction (PCR) using Hoffmann universal primers. Amplified products of expected sizes were purified with the AxyPrep DNA Gel Extraction Kit (Axygen, Hangzhou, China) and sequenced by paired-end Illumina protocols (Beijing Tsingke Biotechnology Co., China). Contigs was assembled using DNASTAR Lasergene v7.1 (DNASTAR Inc., USA).

### Virus purification and titration

Following identification, the viruses were purified using the limited dilution method. Specifically, serial dilutions of the viruses ranging from 10^−3^ to 10^−10^ were prepared, and each dilution was inoculated into five chicken embryos via the allantoic cavity with a volume of 100 μL. After incubation, allantoic fluids were collected exclusively from the HA positive embryos that had been inoculated with the highest dilution of the virus. This purification process was repeated three times to ensure the removal of potential contaminants. Subsequently, PCR assays were conducted to screen for potential co-infections with other viruses, such as infectious bronchitis virus (IBV) and infectious bursal disease virus (IBDV). The purified allantoic fluids were then subjected to large scale propagation in SPF chicken embryos. After harvesting, the samples were immediately stored at −80°C refrigerator to preserve the viral integrity for future experiments. The virus titer was determined as the 50% egg infectious dose (EID_5__0_) using 10-day-old SPF chicken embryos, and calculated according to the Reed and Muench method. This standardized titration procedure was carried out to accurately quantify the viral infectivity, which was essential for subsequent experimental applications.

### Phylogenetic and molecular analysis

Viral sequences were retrieved from the Global Initiative on Sharing All Influenza Data (GISAID) and the National Center for Biotechnology Information (NCBI). The eight gene segments (PB2, PB1, PA, HA, NP, NA, M, and NS) of the retrieved sequences were individually imported into MEGA 11 software. Subsequently, redundant sequences, which could potentially bias the analysis, and incomplete sequences, which might lack essential genetic information, were systematically removed from the dataset. This pre-processing step ensured the quality and integrity of the sequences used for further analysis.

Phylogenetic analysis was conducted based on the amino acid sequences of the remaining gene segments. To construct the phylogenetic trees for all eight segments of the three H3N8 isolates, the Maximum-Likelihood method was employed. This method is a well-established and computationally efficient approach for inferring evolutionary relationships among sequences. The Jones-Taylor-Thornton (JTT) model was used to estimate the genetic distances between sequences which take into account the different rates of transition and transversion mutations. A total of 1000 bootstrap replicates were performed to assess the reliability of the phylogenetic tree topology. Bootstrap values represent the percentage of times a particular node in the tree is supported by the resampled data, providing a measure of the confidence in the inferred evolutionary relationships [26].

### Cells growth kinetics

Madin-Darby canine kidney (MDCK), chicken embryonic fibroblast cells (DF1), and human lung adenocarcinoma epithelial cells (A549) were cultured in Dulbecco’s modified Eagle’s medium (DMEM) supplemented with 10% fetal bovine serum (FBS) and antibiotics (penicillin/streptomycin), and incubated at 37°C under 5% CO_2_. For viral growth kinetics, confluent monolayers in 24-well plates were infected with each virus at a multiplicity of infection (MOI) of 0.01. After 1 hr of adsorption, the inoculum was removed, and cells were washed three times with phosphate-buffered saline (PBS). Subsequently, 500 µL of DMEM containing 1 µg/mL TPCK-trypsin was added to each well. Supernatants were harvested at designated time points (12, 24, 36, 48, 60-hr post infection), and viral titers of all strains were quantified as 50% tissue culture infectious dose (TCID_5__0_) using indirect immunofluorescence assays in MDCK cells. The growth data shown are the average results of three independent experiments [27].

### Receptor binding assay

The solid-phase direct binding assay to test the receptor binding properties of the viruses was described as follows [27]. Briefly, the double diluted 3’SLN-C3-PAA-biot (3’SLN) or 6’SLN-C3-PAA-biot (6’SLN) (GlycoNZ) were coated in ELISA 96-well plates at 4°C overnight, after that PBST containing 5% fat-free milk was added for 8 hr at 4°C, the virus strains containing 6log_2_ hemagglutination units were added and reacted with 3’SLN and 6’SLN, respectively. Subsequently, the virus reacted with specific polyclonal antibody and HRP labeled secondary antibody and the OD450nm was measured. A H9N2 115 strain and A/Puerto Rico/8/1934 (H1N1) (PR8) were selected as α2,3 receptor and α2,6 receptor controls, respectively. Dose-response curves of virus binding to glycol-polymers were analyzed by using a single-site binding algorithm and curve fitting by GraphPad Prism 9.0 to visualize and compare the relative binding profiles among different viral strains. Each value is presented as the mean ± standard deviation (SD) of three independent experiments, each of which was performed in triplicate.

### Heat stability test

The heat stability test was conducted as follows [28]. Test viruses were incubated at 56°C for 5, 15, 30, 60, 90, and 180 min with initial 5log2 HA activity. HA activity was then determined by HA using 1% chicken erythrocytes, and the virus infectivity (TCID_50_) was determined on MDCK cells. All experiments were repeated at least in triplicate.

### Neuraminidase activity assay

Viral NA activity was measured using a fluorescence-based assay [29] with a Neuraminidase Assay Kit (Beyotime Biotechnology, China, catalog number P0306) following the manufacturer’s protocol. Briefly, 70 μL of detection buffer and 10 μL of 2-fold dilution H3N8 virus-containing allantoic fluid (diluted in sterile PBS) were added to a 96-well polystyrene plate. The mixture was gently agitated for 2 min at room temperature. Subsequently, 10 μL of fluorescent NA substrate was added to each well and thoroughly mixed. After incubation at 37°C for 30 min, fluorescence intensity was quantified using a spectrophotometer (BioTek, USA) at excitation/emission wavelengths of 322 nm and 450 nm, respectively. The final NA activity was determined by subtracting the background fluorescence value of the virus-free negative control from the experimental samples.

### Intravenous pathogenicity index (IVPI)

The IVPI was assessed in accordance with the standard operating procedures of the World Health Organization’s international reference laboratory [30] (https://www.woah.org/fileadmin/Home/eng/Health_standards/tahm/A_summry.htm). A total of 40 six-week-old SPF chickens were randomly grouped into 4 groups (10 chickens/group), namely, AH12, JS13, ZJ07, and the PBS mock control. Chickens were then intravenously inoculated with 100 μL of each virus diluted 10-fold in sterile PBS, and monitored daily for 14 days to assess clinical signs, including mortality, morbidity (e.g. lethargy, anorexia), and paralysis. A scoring system was applied as follows: 0 = normal, 1 = sick, 2 = severely sick, and 3 = dead. The IVPI was calculated using the standard formula: IVPI=∑ (daily scores per chicken)/(total number of chickens × observation days).

### Mouse experiments

To evaluate the pathogenicity and virulence of AH12, JS13, and ZJ07 H3N8 viruses in mouse, a total of 104 healthy 6-week-old female BALB/c Mice were randomly grouped into 4 groups, including AH12, JS13, ZJ07, and PBS (mock control), respectively. For the virus group (26 mice per group), each contains four sub-group, which inoculated a dose of 10^3.0^, 10^4.0^, 10^5.0^, and 10^6.0^ EID_50_ of each virus, respectively. Under mixed anesthesia (medetomidine-butorphanol-midazolam), mice were then intranasally inoculated with indicated virus in 50 μL PBS (dose of 10^3.0^, 10,^4.0^ and 10^5.0^ EID_50_ infected 5 mice, dose of 10^6.0^ EID_50_ infected 11 mice). Five mice of each dilution per group were monitored daily for 14 days to record body weight changes and survival rates. The bodyweight dynamics which were recorded as percentage as percentage body weight change relative to baseline. To minimize the potential confounders, two persons who were not aware of this experiment were designed to record the daily weight. Groups of mice were challenged with 10^6^·^0^ EID_5__0_ of the respective virus. Three mice per group were humanely euthanized by cervical dislocation at 3 days and 5 days post-infection (dpi). Nasal turbinate, lung, spleen, kidney, and brain tissues were harvested aseptically for virus titration in embryonated SPF chicken eggs, and viral titers were calculated using the Reed and Muench method. The data analyses were performed using the independent-samples *t* test based on GraphPad Prism software and were shown as the mean ± standard deviation (SD). Animals showing severe disease signs or lose above 25% of their initial weight were euthanized and recorded as having died on the following day. Additionally, lungs from mice euthanized at 5 dpi were fixed in 10% neutral-buffered formalin, embedded in paraffin, and sectioned for hematoxylin and eosin (H&E) staining to evaluate histopathological lesions [29].

### Pathogenicity and transmission experiments in chickens

To evaluate the pathogenicity and virulence of AH12, JS13, and ZJ07 H3N8 virus in SPF chickens, a cohort of 27 six-week-old SPF chickens were randomly allocated into three experimental groups (9 chickens/group). Each group received intranasal inoculation with 10^6.0^EID_5__0_ of respective virus strains suspended in 100 μL sterile PBS. At 5 dpi, three chickens per group were humanely euthanized by intravenous injection of sodium pentobarbital (150 mg/kg) for systematic virological assessment. Tissue specimens including larynx, myocardium, pulmonary parenchyma, hepatic lobe, spleen, kidney, and cecum were aseptically collected for viral titration in 10-day-old SPF chicken embryos and calculated using the Reed and Muench method. Additionally, lungs from chickens euthanized at 5 dpi were fixed in 10% neutral-buffered formalin, embedded in paraffin, and sectioned for hematoxylin and eosin (H&E) staining to evaluate histopathological lesions. The remaining chickens (*n* = 6 per group) underwent clinical evaluation for 14 days. Chickens showing severe disease signs or lose above 25% of their initial weight were euthanized and recorded as having died the following day. Oropharyngeal and cloacal swabs collected at 2-day intervals (0, 2, 4, 6, 8, 10, 12, 14 dpi), the swabs were titrated in 10-day-old SPF chicken embryos and calculated using the Reed and Muench method. Bodyweight dynamics were recorded as the percentage change in body weight relative to baseline. To minimize the potential confounders, two persons who were not aware of this experiment were designed to record the clinical evaluation and daily weight.

To investigate the transmission characteristics of the H3N8 virus among chicken flocks, 36 six-week-old SPF chickens were randomly grouped 3 groups, including AH12, JS13, ZJ07 groups (12 chickens/group). For direct-contact transmission assessment, 6 six-week-old chickens were intranasal inoculated with 10^6.0^EID_5__0_ of each virus in 100 µL PBS. At 24 hpi, three infected chickens and three naïve chickens per group as sentinel chickens were cohoused in one isolator (direct-contact group, DC). Concurrently, airborne transmission potential was assessed using a modified setup: three naïve chickens per group were individually housed in each cage encircling the cage of the three inoculated chickens (airborne group, AB), cages were spaced 15 cm apart at least to ensure shared airspace without physical contact. Oropharyngeal and cloacal swabs were collected at 2-day intervals from 0 to 14 dpi. Viral titers were quantified through allantoic cavity inoculation of 10-day-old SPF chicken embryos, and calculated using the Reed and Muench method. The serum samples collected at 21 dpi were analyzed for hemagglutination inhibition (HI) antibodies using homologous antigens [28].

### Statement of adherence to ARRIVE guidelines

In this study, we followed the Animal Research: Reporting of In Vivo Experiments (ARRIVE) guidelines to ensure transparency and integrity in the design, execution, and reporting of our experiments. The ARRIVE guidelines are intended to improve the quality of scientific research, to ensure that experimental animal use is properly justified, and to promote the reliability and reproducibility of scientific discoveries. We have adhered to ARRIVE guidelines and uploaded a completed checklist as supplementary files.

## Results

### Isolation and characterization of the H3N8 viruses

Three H3N8 avian influenza viruses (designated AH12, JS13, and ZJ07), isolated from seemingly healthy chickens in three Chinese provinces in 2022, were selected for further characterization. Viral titers determined by the Reed–Muench method revealed EID_50_ ranging from 9.25 to 9.5 Log_10_ EID_50_/mL, with HA titers of 512 to 2048 HAU per 25 μL. HI assays demonstrated strict serological specificity, all isolates reacted exclusively with H3-subtype avian influenza antisera (HI titers ≥128), showing no cross-reactivity with antisera against other influenza subtypes (H5, H7, and H9), Newcastle disease virus (NDV), or egg drop syndrome virus (EDS-76) (S1 Table, supporting information). These results validate the successful isolation of H3N8 viruses from asymptomatic chickens in eastern China, characterized by high titers, robust HA activity, and subtype-specific antigenicity. The isolated viruses demonstrated exceptionally efficient replication kinetics and potent infectivity in chicken embryos, underscoring their strong adaptability to avian hosts.

### Genetic and phylogenetic characterization of the H3N8 viruses

To elucidate the evolutionary origins and genetic relationships of the representative H3N8 isolates, whole-genome sequencing (S2 Table, supporting information) and maximum-likelihood phylogenetic analysis (MEGA 12) were performed. Phylogenetic trees revealed distinct clustering patterns: the HA and neuraminidase (NA) genes formed a monophyletic clade (bootstrap ≥98%) with 97.4–100% nucleotide identity to the human-origin H3N8 viruses (e.g. A/Henan/4-14CNIC/2022, A/Guangdong/ZS-23SF005/2023), and the HA genes were diverging significantly from swine/canine H3N2 lineages (Figure 1(A,B)).

**Figure 1.**
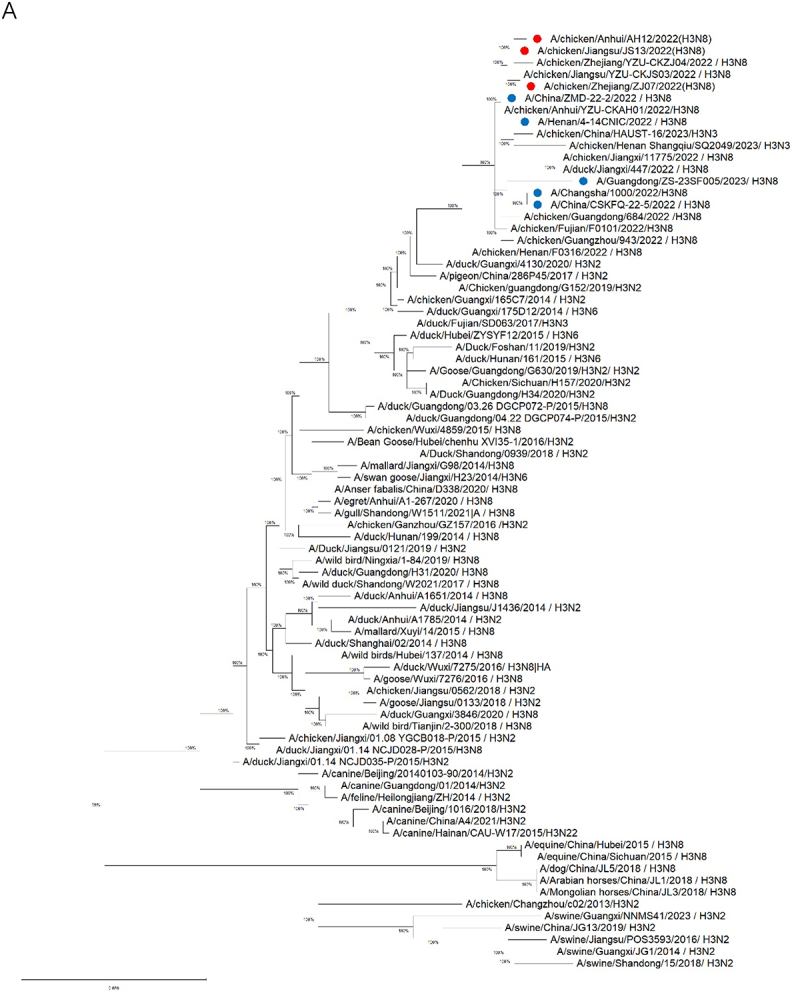
Phylogenetic analysis of the genomes of the H3N8 viruses. Phylogenetic trees were constructed using MEGA software (Version 12) with the maximum-likelihood method. Bootstrap value of 1000 was used to estimate the statistical reliability of clades, and values higher than 90 were automatically shown above or below the branch. The viruses with red dot are used in this study, and viruses with blue dot are human isolates. (A): Phylogenetic tree of HA gene of the H3N8 AIVs (B): Phylogenetic tree of NA gene of the H3N8 AIVs.

**Figure 1.**
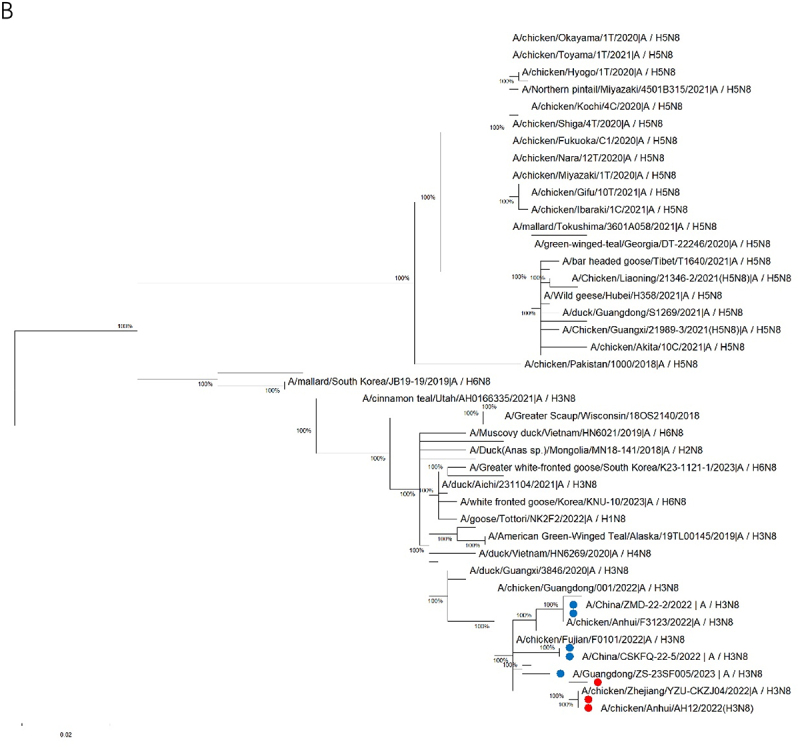
(Continue).

Evolutionary analysis identified six internal genes (PB2, PB1, PA, NP, M, and NS) originated from the H9N2 viruses, sharing >98% homology with human-infecting H3N8 variants (e.g. A/Changsha/1000/2022) (S2 Figure A-F, supporting information). Notably, HA and PB2 genes shared 96.8–99.1% nucleotide identity with an emergent triple-reassortant H3N3 strain derived from H3N8, H9N2, and H10N3 virus, demonstrating recurrent inter-subtype reassortment in poultry reservoirs. All genome sequences deposited in NCBI GenBank, the accession number for the H3N8 sequences used in this study are: PQ676161, PQ676075, PQ676070, PQ676061, PQ625896, PQ625894, PQ625892, and PQ625891 for A/chicken/Zhejiang/ZJ07/2022(H3N8); PQ680196, PQ680195, PQ680193, PQ680178, PQ676189, PQ625911, PQ625909, and PQ625902 for A/chicken/Anhui/AH12/2022(H3N8); PQ681295, PQ681294, PQ681293, PQ681291, PQ681288, PQ681287, PQ625916, and PQ625915 for A/chicken/Jiangsu/JS13/2022(H3N8).

### Receptor binding properties of the H3N8 viruses

**Figure 2.**
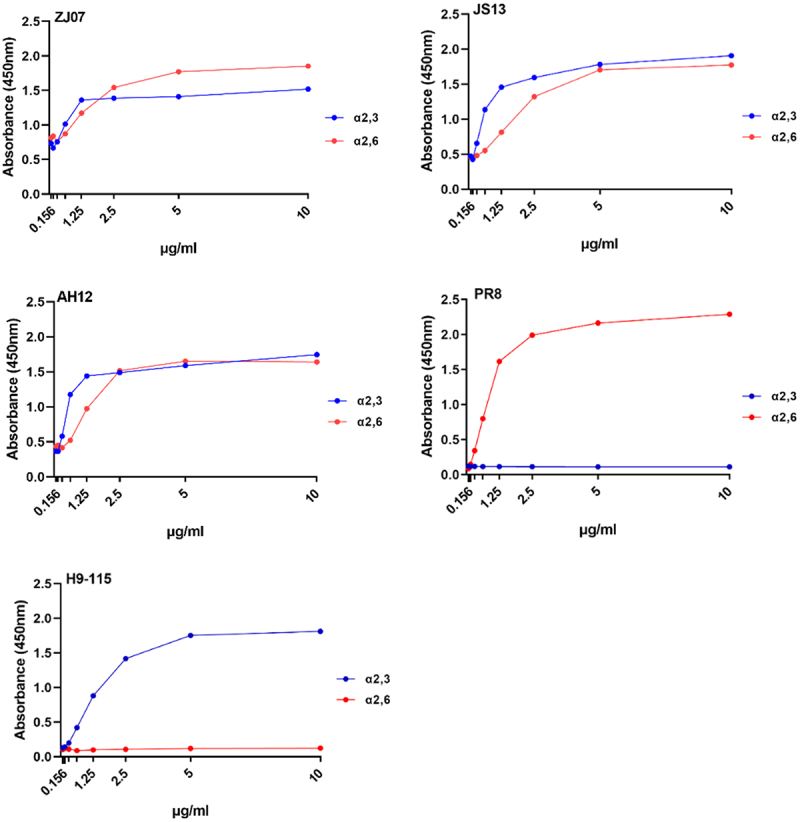
Receptor-binding properties of the H3N8 viruses. The receptor binding of the H3N8 viruses was determined using various concentrations of sialic acid conjugated to biotinylated sialylglycopolymers (3′SLN and 6′SLN) via direct solid-phase binding assays, a H9N2 AIV isolate (115 strain) and A/Puerto Rico/8/1934 (H1N1) (PR8) were selected as α2,3 receptor and α2,6 receptor controls, respectively.

To assess the host adaptation potential of the H3N8 isolates, receptor binding specificity was analyzed via solid-phase binding assays using synthetic glycans mimicking avian (α2,3-linked sialic acid) and human (α2,6-linked sialic acid) receptors. All isolates displayed dual receptor tropism, binding to both α2,3 and α2,6 receptors – a hallmark of human-adapted influenza variants, and exhibited comparable binding affinity for both type receptors (Figure 2).

### Molecular characteristics of the H3N8 viruses

We examined the molecular properties of amino acid sequences to assess the risk to poultry and mammals. The results showed that the sequences have the same amino acid motif PEKQTR↓GLF at the cleavage site of the HA protein which is the same as those human-originated H3N8 viruses, indicating that they belong to low pathogenic avian influenza virus. All three isolates contained identical conserved residues (98 Y, 153 W, 183 H, 190 E, 194 L, and 195 Y; H3 numbering) in the HA receptor-binding domain (RBD). Critical residues determining receptor-binding specificity (222 W, 226 Q, 227 S, and 228 G), which are known to mediate preferential binding to α2,3-linked or α-2,6-linked sialic acids in H3 subtype viruses [14,31,32], were conserved in all isolates and matched those in five human H3N8 isolates (S3 Table, supporting information). All isolates retained the avian-type receptor-binding motif (Q226/G228) in HA and lacked canonical mammalian adaptation markers (E627K, D701N, and T702K) in PB2. However, our analysis identified multiple putative mammalian adaptation signatures, including HA-225 G, PB2-588 V, PB1-473 V, PA-295P, PA-383D, PA-409N, PA-476A, and PA-630E [33–36]. Additionally, we detected several substitutions associated with enhanced mammalian pathogenicity: PB2-292 V, PB2-598I, PB1-368 V, PB1-622 G, PA-356 R, M1-30D, M1-43 M, M1-215A, NS1-42S, NS1-172K, and NS1-205S. These observations suggest concurrent evolutionary trajectories for avian receptor maintenance and potential mammalian adaptation through alternative molecular pathways. Comparative analysis revealed distinct amino acid variations among the isolates. The HA proteins of AH12 and JS13 strains contained R62, I70, and I363, while ZJ07 showed G62, M70, and V363 substitutions. Additional polymorphisms included G78/D193 in AH12 versus V78/N193 in ZJ07 and JS13. All strains carried the S31N substitution in the M2 ion channel, a known marker of amantadine resistance [21] (Table 1). Notably, all three isolates exhibited a unique N-glycosylation pattern in HA, with eight potential glycosylation sites (positions 22, 38, 53, 145, 165, 170, 285, and 483; H3 numbering) (S4 Table, supporting information).

**Table 1.** Molecular characteristics of H3N8 virus.

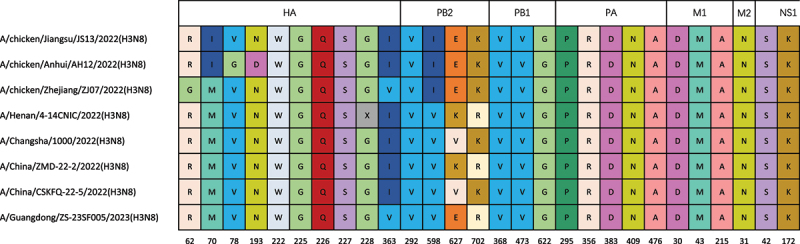

### Thermal stability of the H3N8 viruses

**Figure 3.**
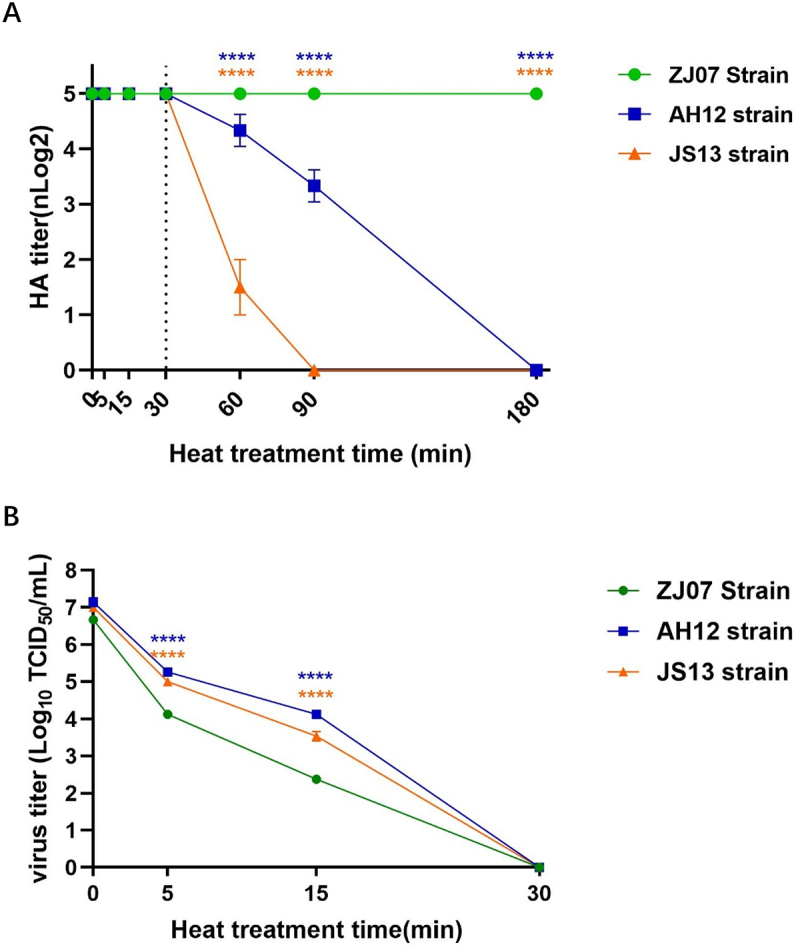
Biological activity of the H3N8 viruses. (A) Thermal stability of the H3N8 viruses were conducted by incubating viral suspensions at 56°C for predetermined durations (5, 15, 30, 60, 90, 180 minutes). HA activity was assayed using 1% chicken erythrocytes, and (B) viral infectivity was evaluated through TCID_50_ determination in MDCK cells. Data were analyzed by two-way ANOVA with multiple comparisons. Asterisks indicate significant differences (* = *p* < 0.05, ** = *p* < 0.01, *** = *p* < 0.001, **** = *p* < 0.0001); ns = no significant differences. The blue asterisks represent the comparison between the ZJ07 and AH12 strains; the orange asterisks represent the comparison between the ZJ07 and JS13 strains.

Viral thermal stability was assessed by incubating suspensions at 56°C for specified durations (5, 15, 30, 60, 90, and 180 min). Hemagglutination (HA) activity was measured using 1% chicken erythrocytes, and infectivity was determined by TCID50 assay in MDCK cells. Time-course analysis revealed strain-specific thermal resistance profiles. The JS13 strain showed an initial HA titer reduction (3.5 log_2_ decrease) after 30 min, with complete inactivation by 90 min. In contrast, AH12 maintained detectable HA activity until 90 min (4.5 log_2_ at 60 min; 3.5 log_2_ at 90 min), with complete loss observed at 180 min. Notably, ZJ07 retained full HA activity throughout the 180-min exposure (Figure 3(A)). All strains completely lost infectivity (TCID_50_ undetectable) after 30 min exposure (Figure 3(B)). These results demonstrate significant inter-strain variation in H3N8 virion heat resistance at 56°C, with preserved HA activity despite rapid infectivity loss.

### Neuraminidase activity of the H3N8 viruses

Viral neuraminidase (NA) activity was quantified using a fluorescence-based assay. Following 30-min incubation at 37°C, fluorescence intensity was measured at excitation/emission wavelengths of 322 and 450 nm, respectively. Net NA activity was determined by subtracting the background fluorescence values of virus-free controls from experimental samples. All three H3N8 isolates, ranging from the stock solution to a 1:64 dilution, demonstrated significantly higher fluorescence intensities compared to the neuraminidase-positive control (provided by the Neuraminidase Assay Kit, Beyotime Biotechnology, catalog number P0306) (*p* < 0.0001). Strikingly, upon further dilution to 1:128, the JS13 isolate maintained superior fluorescence intensity among all tested samples, indicating its enhanced enzymatic stability under low viral load conditions (S1 Fig, supporting information).

### Replication kinetics of the H3N8 viruses in avian and mammalian cells

**Figure 4.**
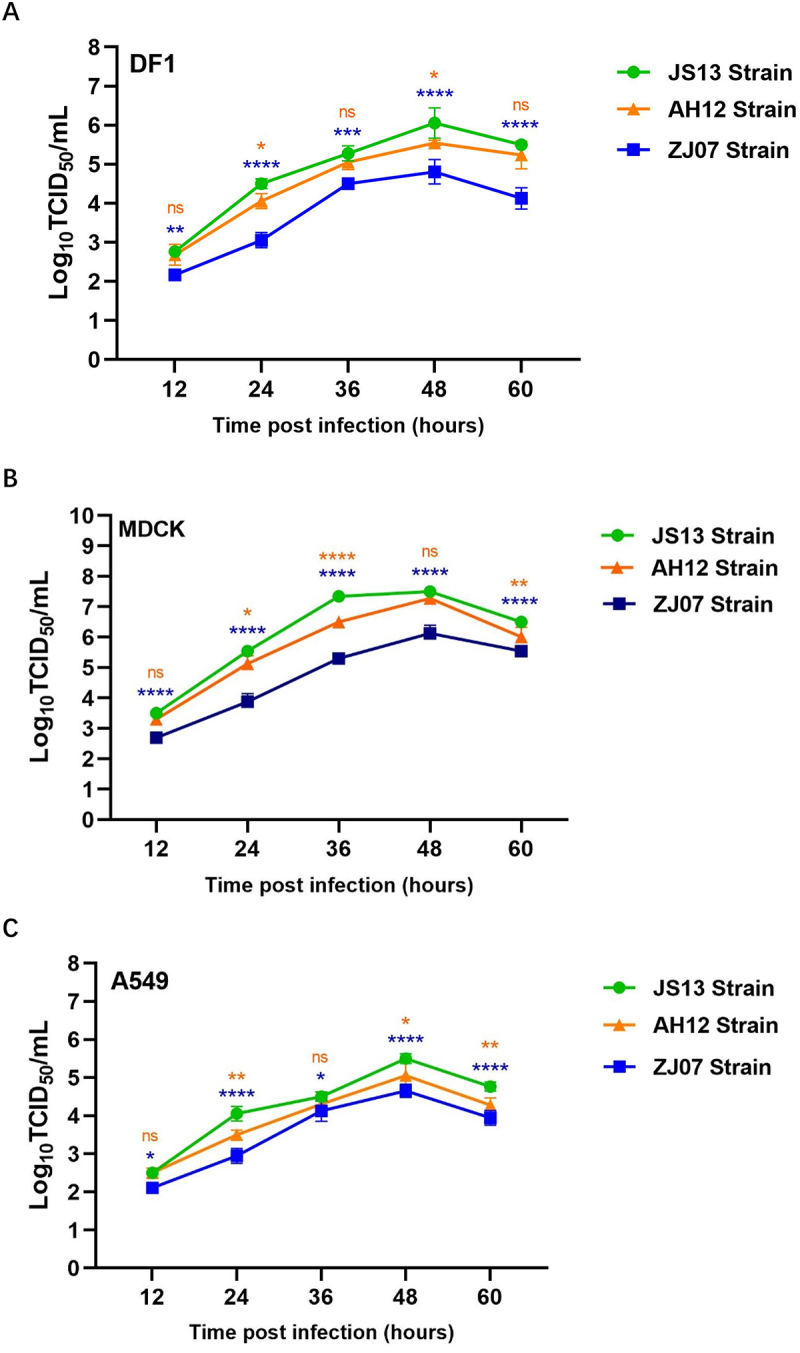
Growth kinetics of the H3N8 viruses in (A) DF1, (B) MDCK and (C) A549 cells. Cells were infected with each virus at an MOI of 0.01. Supernatant samples were collected at 12, 24, 36, 48 and 60 hpi, and viral titers were measured in MDCK cells, respectively. Data were analyzed by two-way ANOVA with multiple comparisons. Asterisks indicate significant differences (* = *p* < 0.05, ** = p < 0.01, *** = *p* < 0.001, **** = *p* < 0.0001); ns = no significant differences. The orange asterisks represent the comparison between the JS13 and AH12 strains; the blue asterisks represent the comparison between the JS13 and ZJ07 strains.

To evaluate the host adaptability of H3N8 viruses, replication kinetics of three isolates (AH12, JS13, ZJ07) were systematically compared in DF-1 (avian), MDCK (canine), and A549 (human) cell lines. Viral growth curves generated at a multiplicity of infection (MOI) of 0.01 revealed strain-specific and host-dependent variations, all isolates reached peak titers (4.65~7.5 Log_10_TCID_50_/mL) by 48 hpi across all cell types, indicating the H3N8 viruses possess cross-species replicative capacity in vitro, the efficiency of which is influenced by both viral genetic determinants and host cell tropism. While JS13 and AH12 exhibited comparable replication efficiency, ZJ07 displayed markedly attenuated growth kinetics, with statistically significant reductions (0.47~2.2 Log_10_TCID_50_/mL) observed at all time points from 24 to 60 hpi (Figure 4(A-C)). Notably, MDCK cells (Figure 4(B)) supported significantly higher viral yields than DF-1 (Figure 4(A)) and A549 cells (Figure 4(C)), with inter-strain differences amplified in mammalian hosts (MDCK vs. A549: Δ1.48–2.22 Log_10_TCID_50_/mL at peak). These data demonstrate that while H3N8 viruses retain cross-species replicative capacity, their fitness is influenced by both viral genetic determinants and host cell tropism.

### Replication and virulence of the H3N8 viruses in mice

**Figure 5.**
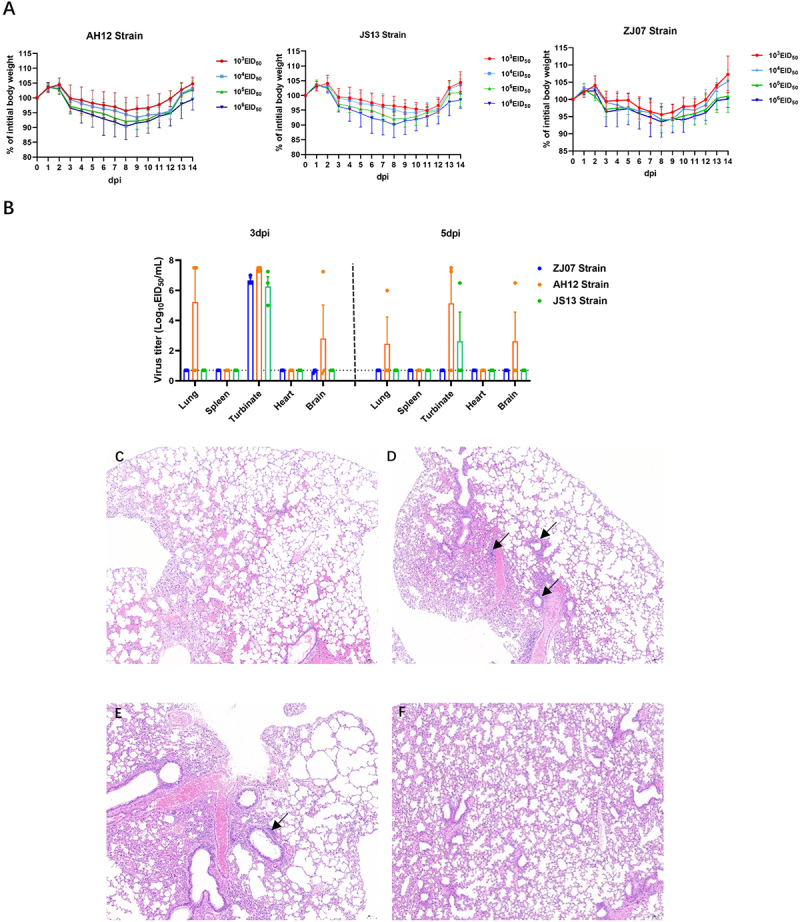
Replication and virulence of the H3N8 viruses in mice. Six-week-old BALB/c mice were infected intranasally with 10^3^-10^6^ECID_50_ of each virus. (A) Percentage of bodyweight change of mice infected with each virus. (B) The viral titers of the nasal turbinate, lung, spleen, kidney and brain tissues of the infected mice collected at 3 and 5 dpi were measured in embryonated SPF chicken eggs. (C-F) H&E staining of lung tissues from mice inoculated with (C) ZJ07, (D) AH12, (E) JS13, or (F) control.

Groups of 6-week-old female BALB/c mice intranasally inoculated with 10^3^ ~10^6^ EID_5__0_ of H3N8 viruses exhibited non-lethal infections with strain-dependent pathogenicity. All mice survived the 14-day observation period, displaying transient clinical morbidity characterized by peak body weight loss (4.4%~10%) at 8 dpi, followed by gradual recovery (Figure 5(A)). Viral replication analysis revealed distinct organ tropism patterns among strains. At 3 dpi, all strains replicated efficiently in the nasal turbinate of all mice (*n* = 9). Notably, AH12 uniquely demonstrated pulmonary and brain tropism. By 5 dpi, ZJ07 was cleared from all organs, whereas JS13 persisted in the nasal turbinate, and only AH12 could be detected in nasal turbinate, brain, and lungs. No infectious viral particles were detected in the spleen or heart at any timepoint (Figure 5(B)). Histopathological examination of lung tissues revealed no significant parenchymal damage; however, mild perivascular lymphocyte infiltration was observed in AH12 and JS13 strain infected mice (Figure 5(C-F)).

### Pathogenicity and virulence of the H3N8 viruses in chickens

**Figure 6.**
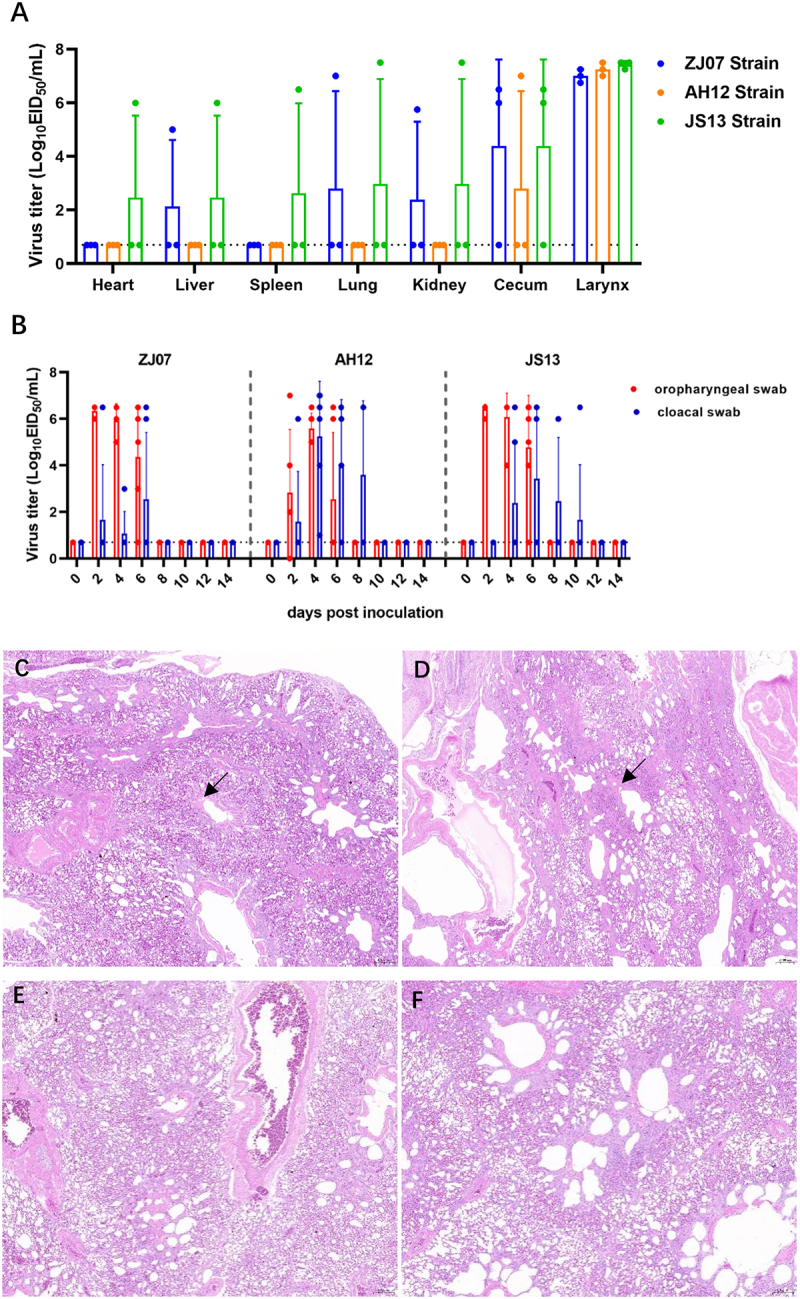
Replication and pathogenicity of the H3N8 viruses in chickens. Six-week-old SPF chickens were intranasal inoculated with 10^6^ EID_5__0_ of each virus. (A) Tissue specimens including larynx, myocardium, pulmonary parenchyma, hepatic lobe, spleen, kidney, and cecum were aseptically collected at 5 dpi. For viral titration in 10-day-old SPF chicken embryos. (B) Oropharyngeal and cloacal swabs were collected from the chickens at the indicated time points, and the viruses were titrated in embryonated SPF chicken eggs. H&E staining of lung tissues from chickens inoculated with (C) ZJ07, (D) AH12, (E) JS13, or (F) control.

The H3N8 viruses characterized in this study were isolated from asymptomatic poultry during active surveillance. Although H3N8 has been reported in systemic organs [14,23] or restricted to respiratory tracts [21], we systematically evaluated their pathogenicity in SPF chickens through experimental inoculation. Viral replication was detected in the larynx and cecum across all strains. ZJ07 and JS13 additionally showed tropism for the liver, lungs, and kidneys, with JS13 exhibited the broadest distribution (including spleen and heart). In contrast, AH12 was detected only in larynx and cecal samples (Figure 6(A)). Viral shedding kinetics varied significantly, two-thirds (67%) of AH12-inoculated chickens shedding virus at 2 dpi, reaching 100% by 4 dpi. In contrast, all chickens infected with either ZJ07 or JS13 exhibited shedding from 2 dpi onward. We observed distinct strain-dependent shedding durations: 6 days for ZJ07, 8 days for AH12, and 10 days for JS13 (Figure 6(B)). Notably, despite systemic replication, no chickens exhibited clinical signs, mortality, or significant weight loss during the 14-day observation period. Histopathology revealed no severe lesions, consistent with the low-pathogenicity HA motif. Mild interstitial edema and perivascular lymphocyte infiltration were observed in the lungs (Figure 6(C-F)). These findings demonstrate that H3N8 virus can achieve systemic dissemination in chickens without clinical disease, suggesting host adaptation mechanisms that balance replication efficiency with attenuated virulence.

### Transmission characteristic of the H3N8 viruses in chickens

**Figure 7.**
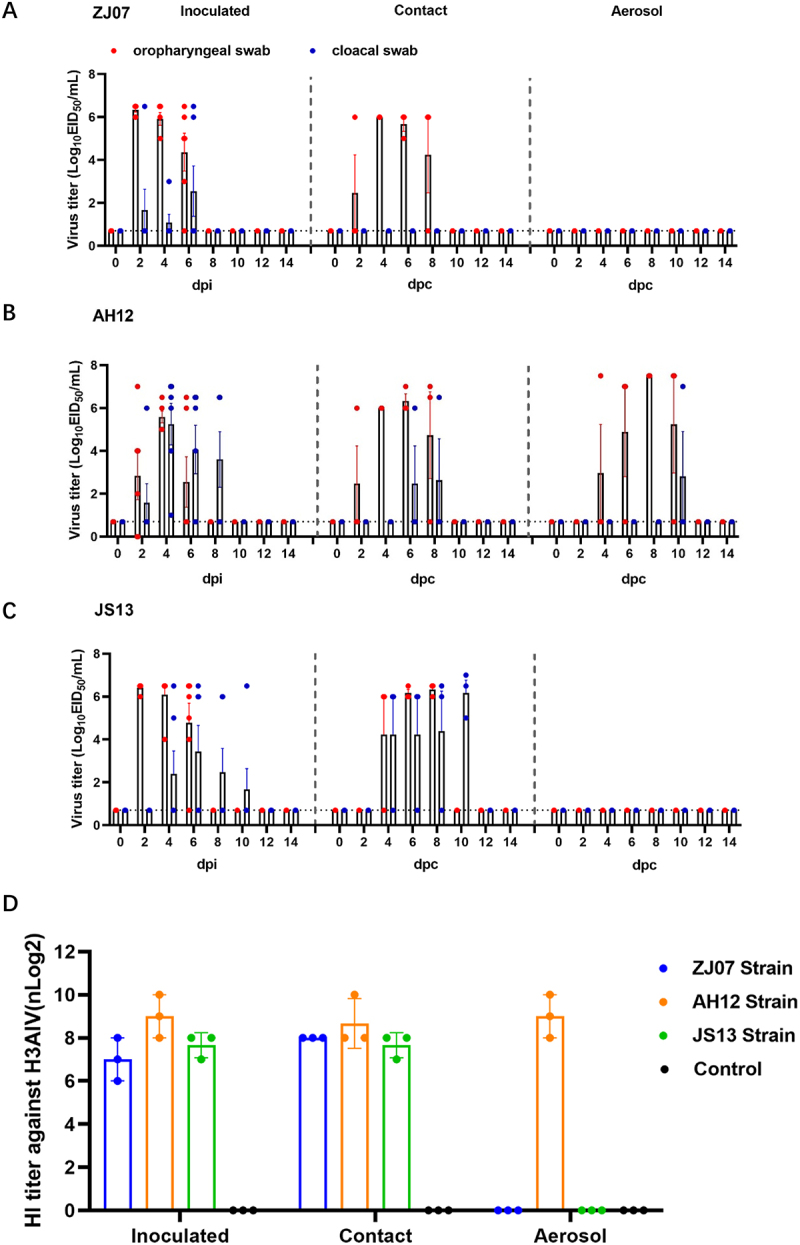
Replication and transmission of the H3N8 viruses in chickens. (A-C) Virus shedding and transmission of the H3N8 viruses in chickens. Oropharyngeal and cloacal swabs were collected at the indicated time points, and the viruses were titrated in embryonated SPF chicken eggs. (D) Serum samples from all chickens were collected at 21 dpi to detect HI antibodies.

To assess transmission potential, seronegative chickens were intranasally inoculated with 10^6^ EID_50_ of each virus. Transmission was evaluated using physical-contact and airborne exposure models, with viral shedding monitored via oropharyngeal and cloacal swabs and seroconversion confirmed at 21 dpi. The AH12 strain showed efficient transmission through both direct-contact and airborne routes (100%). In contrast, ZJ07 and JS13 are transmitted only via physical contact, with no airborne spread detected (Figure 7(A-C)). All transmission-positive chickens began shedding at 2–4 days post-exposed, lasting 6 days. Serological analysis revealed strain- and route-dependent differences, physical-contact groups seroconverted universally, whereas airborne transmission induced detectable antibodies only in AH12-exposed chickens (Figure 7(D)). These findings demonstrate distinct transmission fitness among H3N8 variants, with AH12 uniquely capable of airborne spread – a significant factor for cross-species transmission risk at avian-mammal interfaces.

## Discussion

The isolation of H3N8 avian influenza viruses (AIVs) from asymptomatic chickens in eastern China reveals a concerning ecological dynamic of silent viral circulation with low pathogenicity (HA cleavage motif: PEKQTR↓GLF) [23]. This phenomenon, occurring at the avian-human-livestock interface, provides a persistent source for viral evolution. These viruses exhibit high replication competence and hemagglutination (HA) activity, suggesting efficient avian adaptation. Their genomic architecture – featuring HA/NA genes closely related to human-like H3N8 viruses (e.g. A/Henan/4-14CNIC/2022) and internal genes from enzootic H9N2, with additional homology shared between its HA/PB2 genes and those of emerging H3N3 viruses – highlights the role of reassortment-driven evolution between subtypes [37]. These findings collectively delineate the zoonotic evolution of H3N8 influenza viruses, characterized by a dual-origin genomic architecture where surface glycoproteins (HA/NA) align closely with human-adapted strains, while internal genes derive from poultry H9N2 lineages. This study provides clear evidence of cross-subtype reassortment driving adaptive plasticity in avian hosts, posing potential risks for interspecies transmission and pandemic emergence.

A molecular comparison with human H3N8 strains reveals a concerning degree of pre-adaptation. While retaining avian-type HA residues (Q226/G228), these isolates show enhanced binding to human-type (α2,6-linked) receptors, a trait shared with human-adapted strains that indicates poultry-derived H3N8 viruses possess dual receptor-binding capacity. Notably, this comparison with contemporary human H3N8 strains (e.g. A/Henan/4-14CNIC/2022) reveals convergent evolution in receptor-binding preference, although our avian isolates lack the full suite of canonical mammalian-adaptive markers found in human viruses. Conservation of critical RBD residues (222W, 227S) across avian/human isolates and mammalian-like glycosylation (e.g. HA-285N) further suggest pre-adaptation for spillover. The 193D/N variation in antigenic site B further underscores immune evasion potential. Despite lacking canonical markers (PB2-E627K) [38], these strains harbor mammalian-adaptive substitutions (PB2-588 V, PB1-473 V, PA-409N) and amantadine resistance (M2-S31N) [31]. These findings suggest that the evolutionary trajectory toward human adaptation may be facilitated by preexisting mutations in avian reservoirs, thereby escalating risks of cross-species transmission and underscoring the need for enhanced surveillance of poultry-derived viruses [39].

The distinct phenotypes of JS13 and AH12 provide unique mechanistic insights into cross-species adaptation. The JS13 isolate exhibited exceptional NA stability, maintaining significantly higher activity than controls at low viral loads, and its preferential growth in MDCK cells suggests a mechanism for efficient host-cell release, potentially advantageous during transmission bottlenecks. AH12’s broad murine tropism and thermal resilience further highlight divergent adaptation strategies, potentially centered on entry efficiency and environmental stability. The discordance between thermal stability (e.g. ZJ07 retaining HA activity at 56°C for 3 hr) and rapid infectivity loss highlights complex environmental persistence mechanisms with significant biosecurity implications. This characteristic may enhance the environmental spread of the virus and suggests that different viral components, such as the HA protein, may have distinct stability thresholds, a factor influencing transmission ecology. This persistence, combined with our transmission data, is critical: AH12 demonstrated efficient airborne transmission in chickens – a trait absent in JS13/ZJ07—which correlated with its unique pulmonary replication in mice. The inability of JS13/ZJ07 to achieve airborne spread, despite contact transmission, underscores that different molecular bottlenecks govern these modes. Asymptomatic shedding persisted for 6–10 days, facilitating undetected spread and reinforcing the need for sensitive surveillance and stringent biosecurity to prevent farm-to-farm transmission. AH12’s combined traits (airborne transmission, murine multi-organ tropism, and dual receptor binding) position it as a variant of concern [39]. Silent circulation in poultry, coupled with reassortment-prone genomes, necessitates integrated surveillance at avian-human-livestock interfaces. Thus, enhanced measures, such as extended facility downtime for thorough decontamination and vaccines that reduce shedding, are urgently needed to curb the silent prevalence in chicken flocks.

This study has limitations that point to clear future directions. Our functional analysis is based on three representative isolates, and a broader surveillance is needed to capture the full genetic diversity of H3N8. The precise molecular mechanisms behind AH12’s airborne transmission and JS13’s NA stability remain to be confirmed through reverse genetics and structural studies. Furthermore, evaluating the pathogenicity and transmissibility of these viruses in a ferret model would be a critical next step to assess their pandemic potential more directly [40].

Despite these limitations, our findings elucidate the replication, evolution, pathogenicity, and transmission characteristics of the H3N8 virus isolated in eastern China through *in vitro* and *in vivo* experiments. It is worth noting that the identification of an airborne-transmissible strain (AH12) without canonical mammalian-adaptive markers challenges current risk assessment frameworks and necessitates a shift toward phenotypic screening in addition to genetic marker-based prediction. Prioritizing broad-spectrum H3 vaccine development and ecological surveillance will mitigate spillover risks [41].

## Supplementary Material

Supplementary materials including legends20251211.docx

## Data Availability

The data that support the findings of this study are openly available in Science Data Bank at https://doi.org/10.57760/sciencedb.28715 .
