# Supplementary material for: Virulence and transmission characteristic of H3N8 avian influenza virus circulating in chickens in China
Source: Virulence. 2026 Jan 7;17(1):2613516. doi: 10.1080/21505594.2026.2613516 (PMC12802998; doi:10.1080/21505594.2026.2613516)
Supplement: Supplementary materials including legends20251211.docx [file KVIR_A_2613516_SM0397.docx]

**S1 Figure: Neuraminidase activities of the H3N8 viruses. Viral neuraminidase (NA) activity was quantified using a fluorescence-based assay. Following 30-min incubation at 37°C, fluorescence intensity was measured at excitation/emission wavelengths of 322 and 450 nm, respectively. Net NA activity was determined by subtracting background fluorescence values of virus-free controls from experimental samples.**


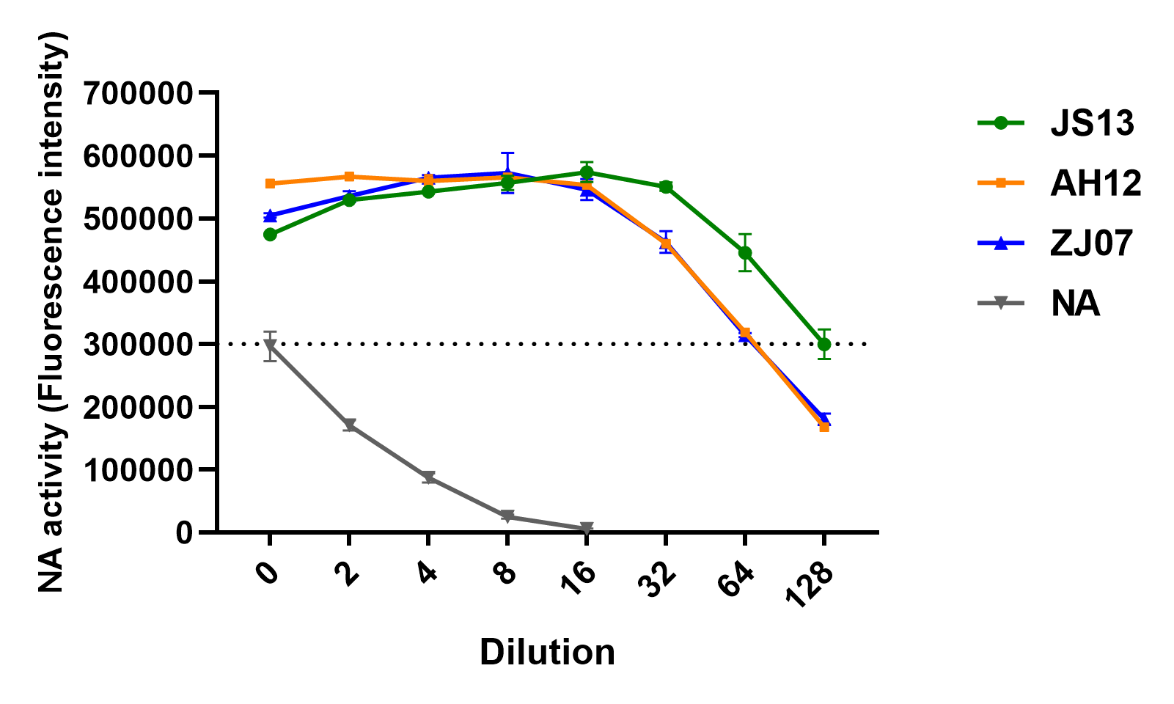


**S2 Figure: Phylogenetic analysis of the genomes of the H3N8 viruses. Phylogenetic trees were constructed using MEGA software (Version 12) with the maximum-likelihood method. Bootstrap value of 1000 was used to estimate the statistical reliability of clades, and values higher than 90 were automatically shown above or below the branch. The viruses with red dot are used in this study, and viruses with blue dot are human isolates.**

**S2 Figure A: Phylogenetic tree of PB2 gene of the H3N8 AIVs**

**
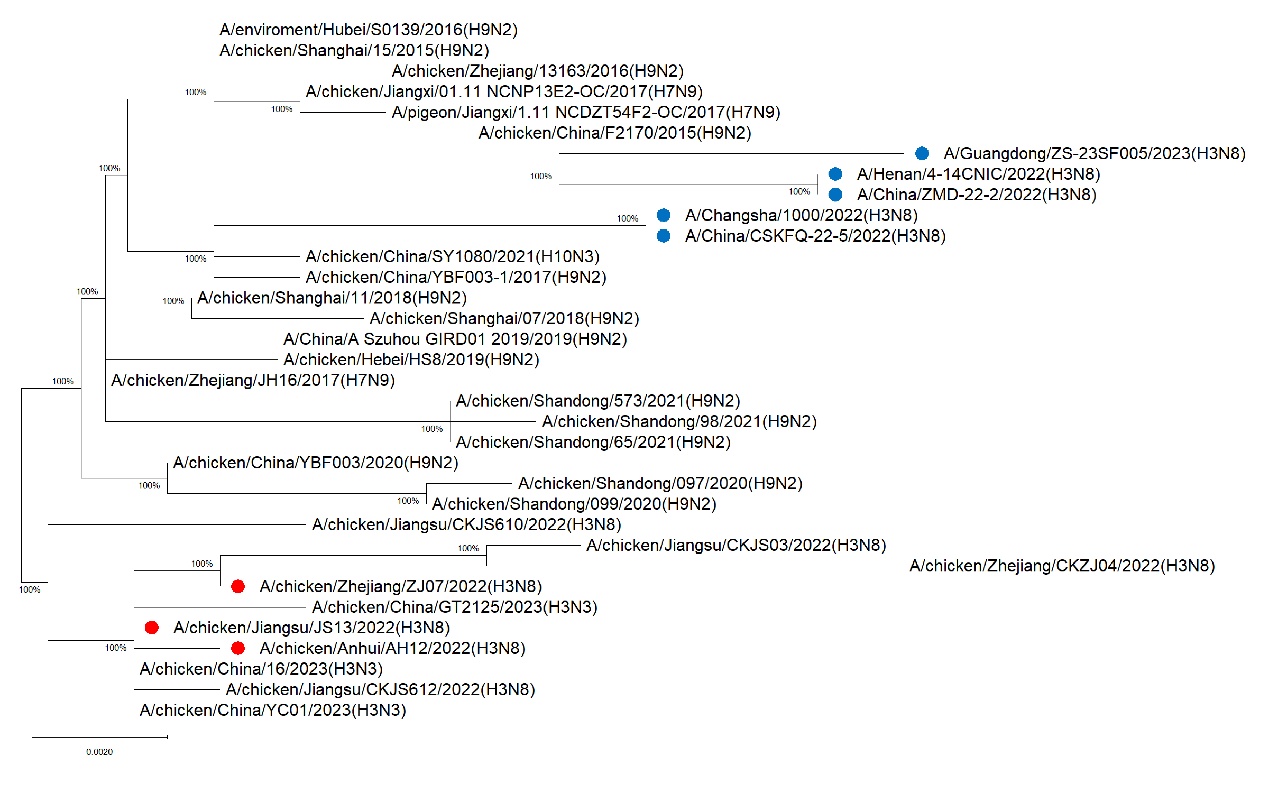
**

**S2 Figure B: Phylogenetic tree of PB1 gene of the H3N8 AIVs**

**
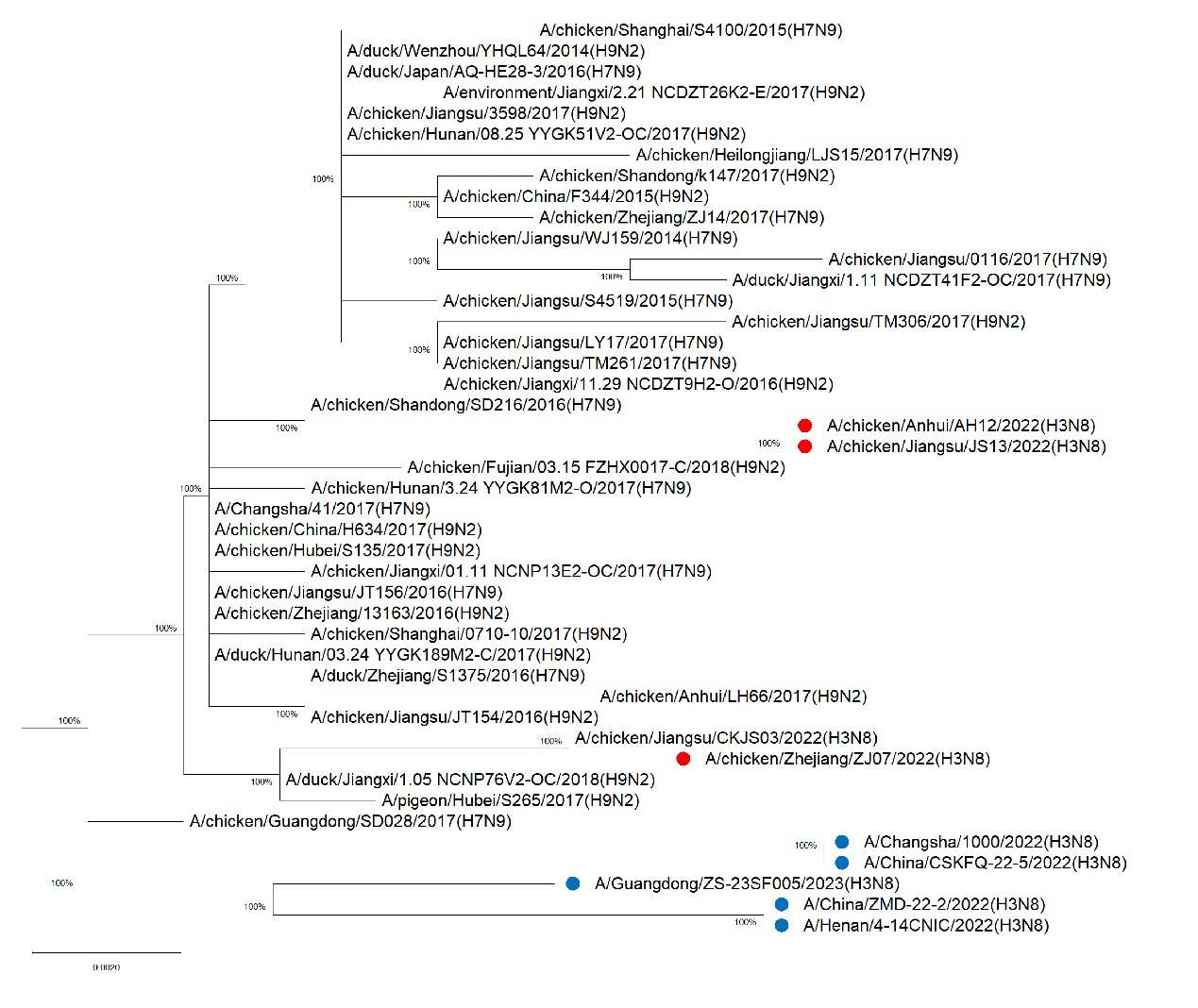
**

**S2 Figure C: Phylogenetic tree of PA gene of the H3N8 AIVs**

**
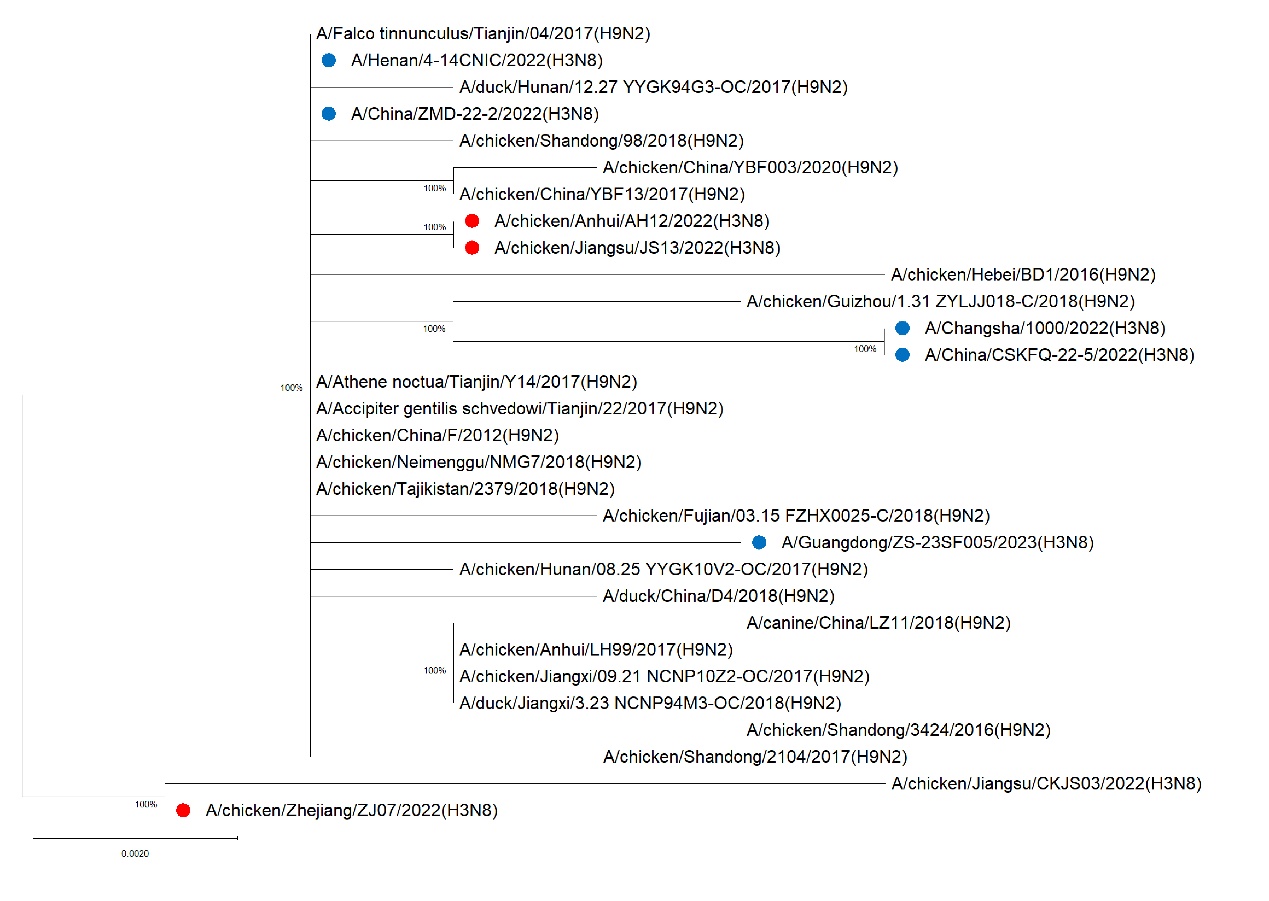
**

**S2 Figure D: Phylogenetic tree of NP gene of the H3N8 AIVs**

**
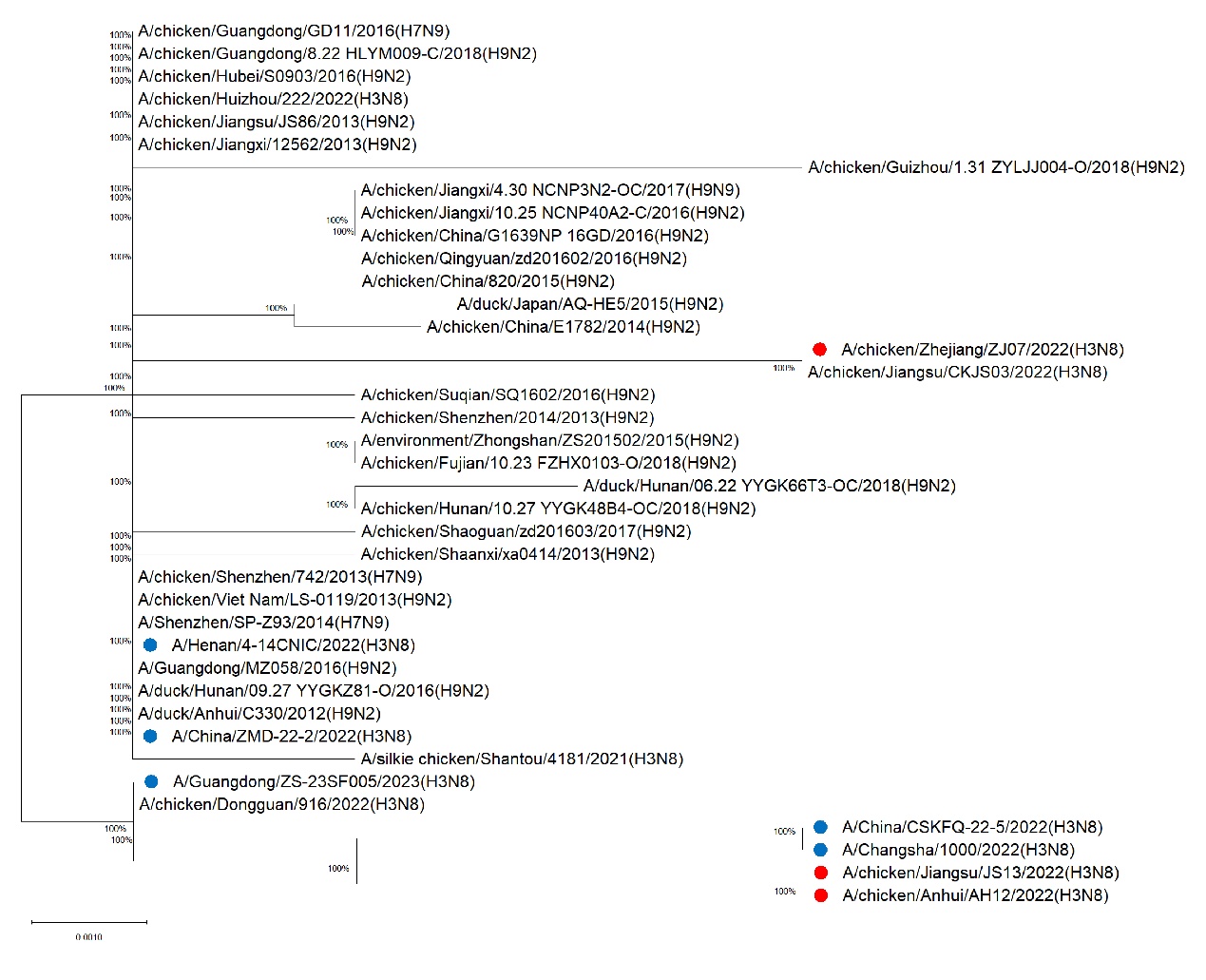
**

**S2 Figure E: Phylogenetic tree of M gene of the H3N8 AIVs**

**
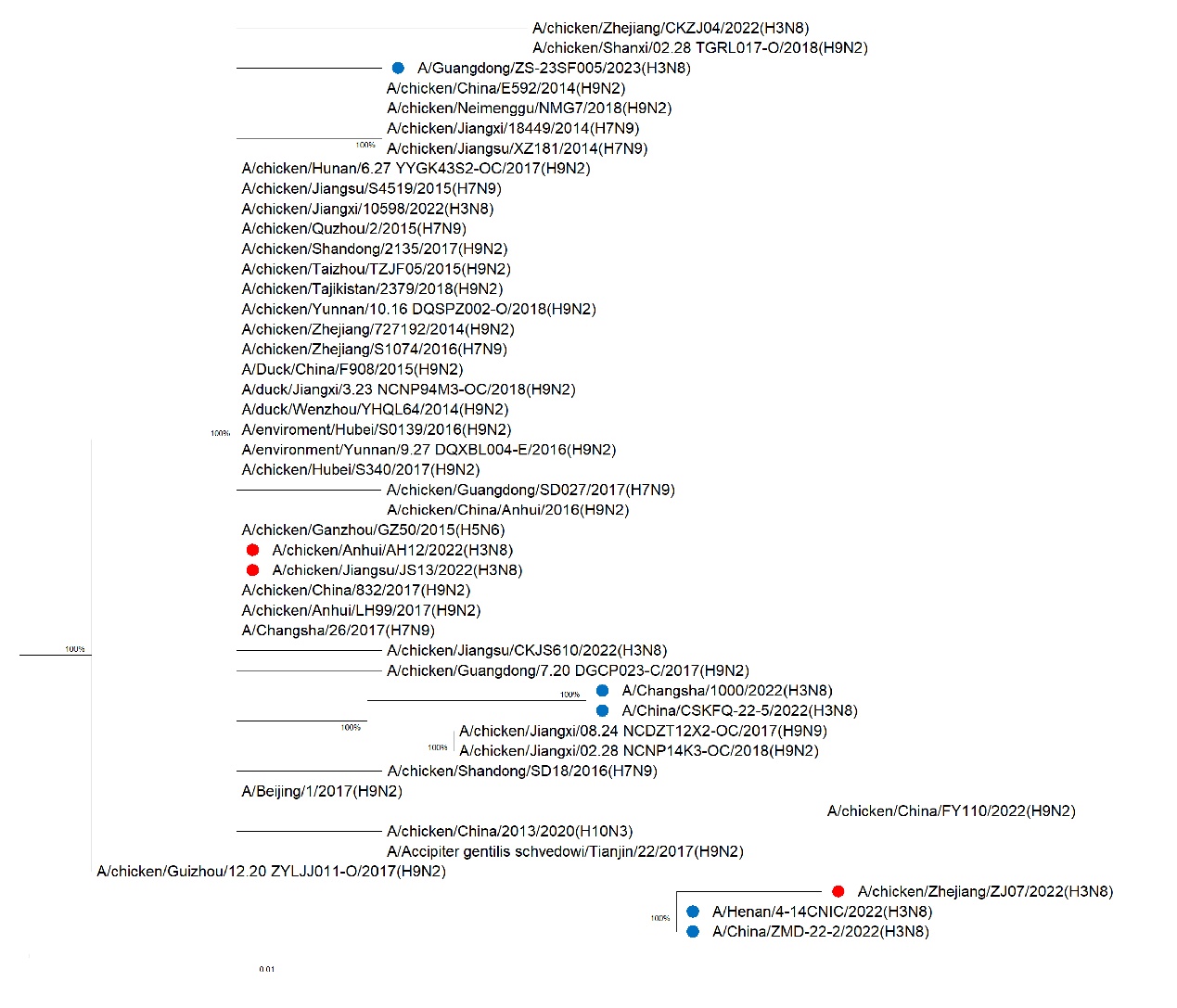
**

**S2 Figure F: Phylogenetic tree of NS gene of the H3N8 AIVs**


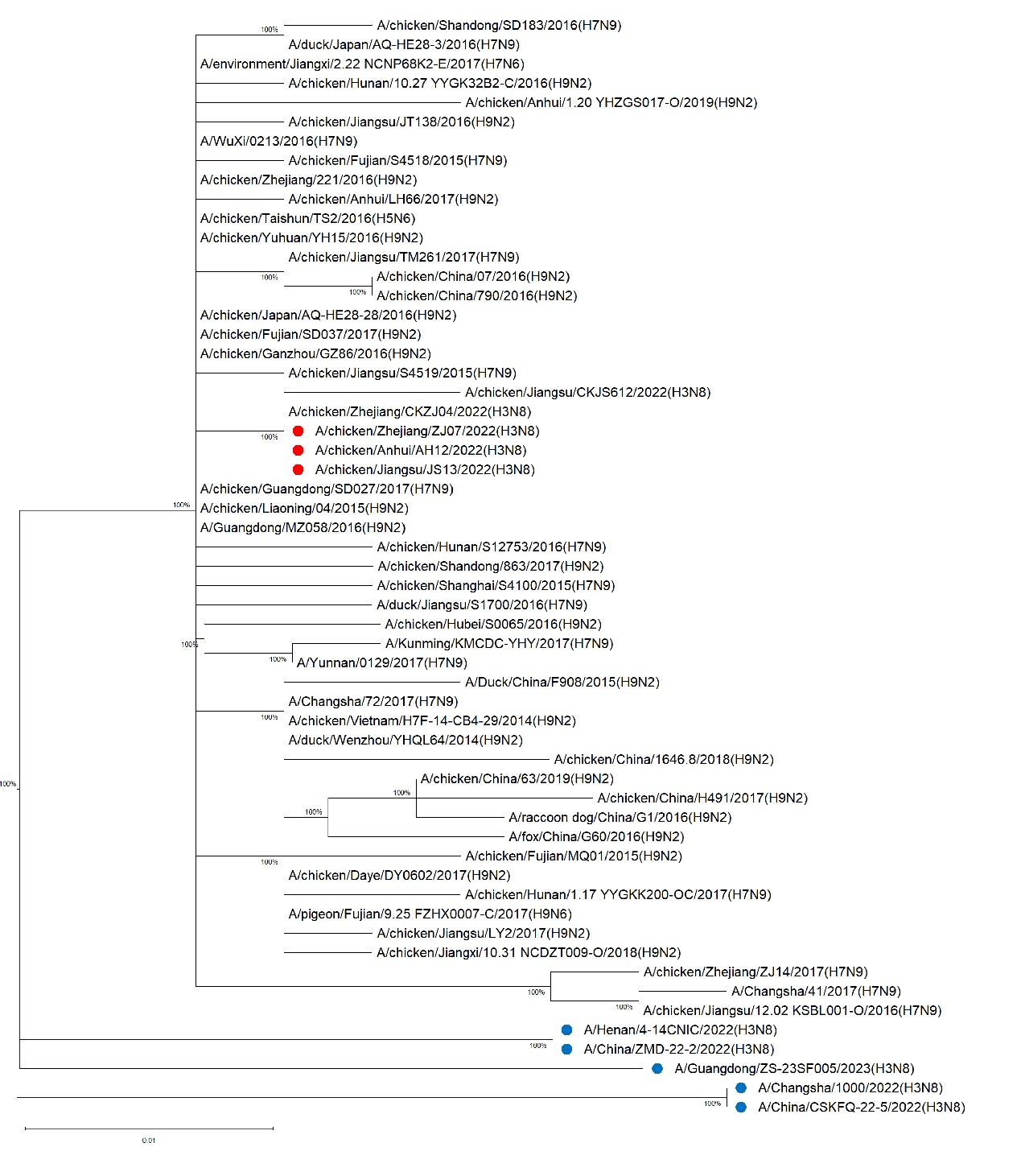


**S1 Table: The EID_50_ and Specificity of the H3N8 avian influenza viruses**

| Virus strain | HA titer (nlog2) | EID_50_/0.1mL | HI titer against positive serum(nlog2) | | | | | | | |
| --- | --- | --- | --- | --- | --- | --- | --- | --- | --- | --- |
|  |  |  | AH12 | JS13 | ZJ07 | H5 | H7 | H9 | NDV | EDS |
| ZJ07 | 9 | 8.5 | 7 | 8 | 8 | 0 | 0 | 0 | 0 | 0 |
| AH12 | 10 | 8.25 | 7 | 7 | 7 | 0 | 0 | 0 | 0 | 0 |
| JS13 | 12 | 8.5 | 7 | 8 | 8 | 0 | 0 | 0 | 0 | 0 |

**S2 Table: Genomic features of the three H3N8 AIVs**

| Segment number | Gene | Abbreviation | Coding sequence (from-to) | Total length/nt | Protein length/ aa |
| --- | --- | --- | --- | --- | --- |
| 1 | Polymerase PB2 | PB2 | 1-2280 | 2280 | 760 |
| 2 | Polymerase PB1 and PB1-F2 protein | PB1 | PB1:1-2274;  PB1-F2: 95-367 | 2274 | PB1:758;  PB1-F2:91 |
| 3 | Polymerase PA and PA-X protein | PA | PA: 1-2151;  PA-X: 1-570, 572-760 | 2151 | PA:717;  PA-X:253 |
| 4 | Hemagglutinin | HA | 1-1701 | 1701 | 567 |
| 5 | Nucleocapsid protein | NP | 1-1497 | 1497 | 499 |
| 6 | Neuraminidase | NA | 1-1413 | 1413 | 471 |
| 7 | Matrix protein 2 and matrix protein 1 | M | M1:1-759;  M2:1-26, 715-982 | 982 | M1:253;  M2:97 |
| 8 | Nuclear export protein and non-structural protein 1 | NS | NS1: 1–654;  NS2: 1–30, 503–838 | 838 | NS1:218;  NS2:122 |

**S3 Table: List of the five human H3N8 strains used in this study**

| Virus Name | Host | Country | Year | Accession Number |
| --- | --- | --- | --- | --- |
| A/Henan/4-14CNIC/2022(H3N8) | Human | China | 2022 | EPI_ISL_12322556 |
| A/Changsha/1000/2022(H3N8) | Human | China | 2022 | EPI_ISL_12703722 |
| A/China/ZMD-22-2/2022(H3N8) | Human | China | 2022 | EPI_ISL_15613648 |
| A/China/CSKFQ-22-5/2022(H3N8) | Human | China | 2022 | EPI_ISL_14870990 |
| A/Guangdong/ZS-23sf005/2023(H3N8) | Human | China | 2023 | EPI_ISL_17464053 |

**S4 Table: Predicted N-glycosylation sites in the HA protein of the three H3N8 avian influenza virus isolates**

| HA Position (H3 Numbering)  Virus Name | Amino Acid Motifs | | | | | | | | |
| --- | --- | --- | --- | --- | --- | --- | --- | --- | --- |
|  | 22 | 38 | 53 | 145 | 165 | 170 | 285 | 483 |  |
| [A/chicken/Zhejiang/ZJ07/2022(H3N8)](https://www.ncbi.nlm.nih.gov/nuccore/PQ676075.1) | NGT | NAT | NNP | NGF | NVT | NNY | NGS | NGT |  |
| [A/chicken/Anhui/AH12/2022(H3N8)](https://www.ncbi.nlm.nih.gov/nuccore/PQ680196.1) | NGT | NAT | NNP | NGF | NVT | NNY | NGS | NGT |  |
| [A/chicken/Jiangsu/JS13/2022(H3N8)](https://www.ncbi.nlm.nih.gov/nuccore/PQ681287.1) | NGT | NAT | NNP | NGF | NVT | NNY | NGS | NGT |  |
